# Supplementary material for: Deletion of wfs1 Impairs Oligodendrocyte Precursor Cells Dorsal Distribution and Myelination Through the wfs1-hmgcs1 Axis in Zebrafish
Source: Biology (Basel). 2026 Aug 16;15(16):1402. doi: 10.3390/biology15161402 (PMC13510151; doi:10.3390/biology15161402)
Supplement: Supplementary file 1 [file biology-15-01402-s001.zip › File S1-Transcriptome sequencing data and differentially expressed genes (DEGs) in wfs1-- zebrafish at 5 dpf/1.GO/wfs1KOvsWT/GSEA/wfs1KO/NUCLEIC_ACID_TRANSPORT/NUCLEIC_ACID_TRANSPORT(GO_0050657).html]

Details for gene set NUCLEIC\_ACID\_TRANSPORT(GO:0050657)[GSEA]


|  || Dataset | deg.sample |
| Phenotype | sample.cls |
| Upregulated in class | wfs1KO |
| GeneSet | NUCLEIC\_ACID\_TRANSPORT(GO:0050657) |
| Enrichment Score (ES) | 0.33185497 |
| Normalized Enrichment Score (NES) | 1.9488583 |
| Nominal p-value | 0.0 |
| FDR q-value | 0.07479778 |
| FWER p-Value | 0.274 |
Table: GSEA Results Summary

  

Fig 1: Enrichment plot: NUCLEIC\_ACID\_TRANSPORT(GO:0050657)
  
Profile of the Running ES Score & Positions of GeneSet Members on the Rank Ordered List

  

| PROBE | DESCRIPTION   (from dataset) | GENE SYMBOL | GENE\_TITLE | RANK IN GENE LIST | RANK METRIC SCORE | RUNNING ES | CORE ENRICHMENT || 1 | 266985 | npm1a |  |  | 83 | 4.783 | 0.1507 | Yes |
| 2 | 393973 | rae1 |  |  | 554 | 2.834 | 0.2267 | Yes |
| 3 | 100005893 | magoh |  |  | 612 | 2.749 | 0.3130 | Yes |
| 4 | 563448 | zgc:193690 |  |  | 1628 | 1.584 | 0.3319 | Yes |
| 5 | 492509 | pcid2 |  |  | 3131 | 1.006 | 0.3169 | No |
| 6 | 100004014 | thoc6 |  |  | 3681 | 0.896 | 0.3283 | No |
| 7 | 768163 | chtopb |  |  | 6462 | 0.540 | 0.2582 | No |
| 8 | 324743 | snupn |  |  | 6714 | 0.518 | 0.2669 | No |
| 9 | 337461 | nup35 |  |  | 6734 | 0.516 | 0.2829 | No |
| 10 | 407727 | slbp |  |  | 8620 | 0.394 | 0.2362 | No |
| 11 | 30471 | qkia |  |  | 9417 | 0.339 | 0.2220 | No |
| 12 | 30967 | igf2bp3 |  |  | 10569 | 0.268 | 0.1945 | No |
| 13 | 403025 | casc3 |  |  | 10662 | 0.263 | 0.2000 | No |
| 14 | 445231 | nup85 |  |  | 10935 | 0.248 | 0.1994 | No |
| 15 | 436971 | tomm20b |  |  | 10957 | 0.247 | 0.2066 | No |
| 16 | 406510 | nup58 |  |  | 11164 | 0.235 | 0.2076 | No |
| 17 | 558918 | slbp2 |  |  | 11287 | 0.228 | 0.2111 | No |
| 18 | 568298 | pom121 |  |  | 11330 | 0.226 | 0.2170 | No |
| 19 | 570158 | alkbh5 |  |  | 11439 | 0.220 | 0.2207 | No |
| 20 | 192325 | ncbp2 |  |  | 11875 | 0.197 | 0.2133 | No |
| 21 | 393917 | ddx39b |  |  | 12078 | 0.186 | 0.2129 | No |
| 22 | 100007012 | si:dkeyp-97b10.3 |  |  | 12184 | 0.180 | 0.2154 | No |
| 23 | 100136839 | igf2bp2a |  |  | 12491 | 0.165 | 0.2111 | No |
| 24 | 796833 | sidt2 |  |  | 12798 | 0.150 | 0.2062 | No |
| 25 | 100529857 | nup214 |  |  | 13495 | 0.115 | 0.1881 | No |
| 26 | 335236 | ndc1 |  |  | 13792 | 0.102 | 0.1820 | No |
| 27 | 571089 | tpra |  |  | 14107 | 0.087 | 0.1749 | No |
| 28 | 567234 | lrmp |  |  | 14178 | 0.084 | 0.1754 | No |
| 29 | 445035 | thoc7 |  |  | 14346 | 0.076 | 0.1726 | No |
| 30 | 321491 | chtopa |  |  | 14836 | 0.054 | 0.1590 | No |
| 31 | 323865 | nxf1 |  |  | 14875 | 0.052 | 0.1594 | No |
| 32 | 406546 | eif5a2 |  |  | 14943 | 0.049 | 0.1589 | No |
| 33 | 322969 | nup50 |  |  | 14954 | 0.049 | 0.1601 | No |
| 34 | 777756 | nup98 |  |  | 15327 | 0.031 | 0.1494 | No |
| 35 | 386850 | eif6 |  |  | 15361 | 0.030 | 0.1494 | No |
| 36 | 558395 | xpo1a |  |  | 15460 | 0.024 | 0.1470 | No |
| 37 | 558883 | tprb |  |  | 16254 | -0.009 | 0.1224 | No |
| 38 | 406464 | eif5a |  |  | 16446 | -0.016 | 0.1169 | No |
| 39 | 324551 | zc3h11a |  |  | 17190 | -0.040 | 0.0948 | No |
| 40 | 563392 | nup62l |  |  | 17218 | -0.042 | 0.0953 | No |
| 41 | 286746 | sdad1 |  |  | 17276 | -0.044 | 0.0950 | No |
| 42 | 562350 | nup107 |  |  | 17374 | -0.049 | 0.0935 | No |
| 43 | 566608 | upf3a |  |  | 17388 | -0.049 | 0.0946 | No |
| 44 | 571621 | nup153 |  |  | 17848 | -0.069 | 0.0824 | No |
| 45 | 795398 | ybx1 |  |  | 17951 | -0.073 | 0.0816 | No |
| 46 | 445302 | thoc3 |  |  | 18025 | -0.077 | 0.0817 | No |
| 47 | 394053 | eif4a3 |  |  | 18040 | -0.078 | 0.0838 | No |
| 48 | 327069 | cpsf6 |  |  | 18096 | -0.081 | 0.0846 | No |
| 49 | 393125 | nup155 |  |  | 18319 | -0.090 | 0.0805 | No |
| 50 | 334749 | seh1l |  |  | 18500 | -0.099 | 0.0780 | No |
| 51 | 561138 | xpo1b |  |  | 18716 | -0.109 | 0.0748 | No |
| 52 | 541444 | nmd3 |  |  | 18754 | -0.111 | 0.0772 | No |
| 53 | 393112 | ncbp3 |  |  | 18755 | -0.111 | 0.0808 | No |
| 54 | 445408 | gle1 |  |  | 19522 | -0.144 | 0.0613 | No |
| 55 | 436700 | eny2 |  |  | 19586 | -0.147 | 0.0640 | No |
| 56 | 436587 | ltv1 |  |  | 19725 | -0.154 | 0.0646 | No |
| 57 | 558182 | igf2bp1 |  |  | 19832 | -0.158 | 0.0664 | No |
| 58 | 30572 | ran |  |  | 20453 | -0.185 | 0.0528 | No |
| 59 | 553507 | npm1b |  |  | 20722 | -0.200 | 0.0508 | No |
| 60 | 406491 | nupl2 |  |  | 21063 | -0.219 | 0.0471 | No |
| 61 | 436588 | polr2d |  |  | 21291 | -0.230 | 0.0473 | No |
| 62 | 325064 | thoc5 |  |  | 21379 | -0.235 | 0.0521 | No |
| 63 | 100536140 | poldip3 |  |  | 21465 | -0.239 | 0.0571 | No |
| 64 | 436589 | mcm3ap |  |  | 21641 | -0.248 | 0.0596 | No |
| 65 | 559225 | nup188 |  |  | 21691 | -0.251 | 0.0661 | No |
| 66 | 337399 | rrs1 |  |  | 21833 | -0.258 | 0.0699 | No |
| 67 | 560127 | alyref |  |  | 22014 | -0.268 | 0.0728 | No |
| 68 | 393815 | qki2 |  |  | 22206 | -0.279 | 0.0758 | No |
| 69 | 373077 | thoc1 |  |  | 23005 | -0.328 | 0.0612 | No |
| 70 | 406324 | abce1 |  |  | 23174 | -0.339 | 0.0668 | No |
| 71 | 368417 | thoc2 |  |  | 23234 | -0.343 | 0.0759 | No |
| 72 | 406852 | nup133 |  |  | 23405 | -0.355 | 0.0819 | No |
| 73 | 558020 | xpot |  |  | 23442 | -0.357 | 0.0922 | No |
| 74 | 445490 | phax |  |  | 23901 | -0.386 | 0.0902 | No |
| 75 | 192339 | ddx19 |  |  | 24093 | -0.399 | 0.0970 | No |
| 76 | 751738 | nxt2 |  |  | 24134 | -0.402 | 0.1086 | No |
| 77 | 751719 | zgc:153681 |  |  | 24257 | -0.411 | 0.1179 | No |
| 78 | 322269 | shfm1 |  |  | 24451 | -0.417 | 0.1252 | No |
| 79 | 503767 | rbm8a |  |  | 24495 | -0.419 | 0.1373 | No |
| 80 | 554106 | sarnp |  |  | 24867 | -0.441 | 0.1397 | No |
| 81 | 325550 | ddx39aa |  |  | 25415 | -0.476 | 0.1378 | No |
| 82 | 321744 | nup88 |  |  | 26614 | -0.589 | 0.1190 | No |
| 83 | 323464 | lsg1 |  |  | 27726 | -0.705 | 0.1067 | No |
| 84 | 30172 | nup93 |  |  | 28090 | -0.755 | 0.1195 | No |
Table: GSEA details
[plain text format]

  

Fig 2: NUCLEIC\_ACID\_TRANSPORT(GO:0050657)
  
Blue-Pink O' Gram in the Space of the Analyzed GeneSet

  

Fig 3: NUCLEIC\_ACID\_TRANSPORT(GO:0050657): Random ES distribution
  
Gene set null distribution of ES for
 **NUCLEIC\_ACID\_TRANSPORT(GO:0050657)**
